# Interleukin-9 promotes intestinal barrier injury of sepsis: a translational research

**DOI:** 10.1186/s40560-021-00550-y

**Published:** 2021-05-03

**Authors:** Jia-Kui Sun, Jing Zhou, Xin-Pei Sun, Xiao Shen, Dong-Mei Zhu, Xiang Wang, Su-Ming Zhou, Xin-Wei Mu

**Affiliations:** 1 Department of Geriatrics Intensive Care Unit, The First Affiliated Hospital of Nanjing Medical University (Jiangsu Province People’s Hospital), 300 Guangzhou Road, Nanjing, 210029 Jiangsu Province China; 2 Department of Critical Care Medicine, Nanjing First Hospital, Nanjing Medical University, 68 Changle Road, Nanjing, 210006 Jiangsu Province China; 3 Department of General Office, Productivity Center of Jiangsu Province, 175 Longpan Road, Nanjing, 210042 Jiangsu Province China

**Keywords:** IL-9-producing CD4(+) T cells, IL-9, Imucosal barrier, Sepsis

## Abstract

**Background:**

Sepsis is a life-threatening organ dysfunction caused by a dysregulated host response to infection. Intestinal mucosal barrier injury is one of the important manifestations of sepsis. Interleukin-9 (IL-9) and IL-9-producing CD4(+) T cells were emerging pro-inflammatory mediators with development of intestinal injury. However, it is unclear whether IL-9 is related to the intestinal barrier injury of sepsis.

**Methods:**

To investigate the roles of IL-9-producing CD4(+) T cells and IL-9 in the process of barrier injury in sepsis, serum IL-9-producing CD4(+) T cell percentages, IL-9, and D-lactate levels were measured in septic patients and controls. The markers of barrier function in serum and intestinal tissue were also collected in septic rats. Moreover, the barrier injury degree and survival rate of septic rats were also investigated after increasing or interfering with IL-9 expression.

**Results:**

The serum IL-9-producing CD4(+) T cell percentages, IL-9, and D-lactate levels were significantly higher in septic patients or rats than those in controls. IL-9-producing CD4(+) T cells and IL-9 levels were positively correlated with D-lactate levels and had a high predictive value of 28-day mortality in septic patients. The non-survivors had significantly higher serum T cell percentages, IL-9, and D-lactate levels compared with survivors. In septic rats, IL-9 increased the expression levels of D-lactate, whereas that decreased the expression levels of zonula occludens 1. Moreover, the barrier injury was aggravated or alleviated by increasing or interfering with IL-9 expression, respectively. Survival rate analysis also showed that IL-9 decreased the 14-day survival rate of septic rats.

**Conclusion:**

IL-9 is closely related to intestinal mucosal barrier injury and mortality in sepsis. IL-9 blockade has the potential to improve the barrier injury in sepsis.

**Trial registration:**

The study was registered at ClinicalTrials.gov (ID: NCT03791866, Date: December 2018).

## Background

Sepsis is a life-threatening organ dysfunction caused by a dysregulated host response to infection [1, 2]. Previous studies have indicated that immune imbalance might play an important role in the development of sepsis [3, 4], which is closely associated with the injury of intestinal mucosal barrier in sepsis [5, 6]. Intestinal tract is a main immune organ supplying an original barrier against pathogenic microorganism [3, 5]. Intestinal barrier injury was often caused by an inflammatory reaction, severe trauma, shock, infection, pancreatitis, and other critical illnesses [3, 5]. Accompanying by sepsis, intestinal epithelial cell damaged, mucosal permeability increased, intestinal flora translocated, and then further intestinal original infection developed [3, 5]. Therefore, intestinal dysfunction is considered as “motor of multiple organ failure.” However, the precise mechanisms of intestinal barrier damage caused by sepsis are still unclear.

In addition to the conventional T helper (Th) 1/Th2 and Th17/T regulatory (Treg) lymphocytes, a latest described Th subset, IL-9-producing CD4(+) T helper cells (Th9), was reported to induce infection, tumor, asthma, inflammatory bowel disease, and other allergic or autoimmune diseases mainly through the interleukin-9 (IL-9) pathway [7–10]. Th9 cells are mainly derived from naïve T cells under the combined stimulation of IL-4 and TGF-β, which are characterized by the secretion of IL-9 and IL-10 [7, 10]. Although IL-9 was also produced by other T helper cells, the Th9 cells were confirmed to be the main cells secreting IL-9 [10]. IL-9 has biological effects on various immune cell types that are involved in the development of inflammation by Janus kinase-signal transduction and transcription activation [11, 12]. Nalleweg et al. [7] found that IL-9 receptor was overexpressed on gut epithelial cells, and the IL-9 level was significantly correlated with intestinal local inflammation in ulcerative colitis. Accordingly, IL-9-producing CD4(+) T cells and IL-9 appear to be new therapeutic targets in allergic or autoimmune diseases [12–15].

Sepsis is characterized by acute severe systemic inflammation and infection, which is distinct from the chronic local inflammation in autoimmune diseases. It is unclear whether IL-9-producing CD4(+) T cells and IL-9 are related to the acute injury of the intestinal mucosal barrier in sepsis. The purpose of this study was to investigate the relationships between IL-9-producing CD4(+) T cells/IL-9 and intestinal barrier injury in clinical septic patients and then verify the roles of IL-9 in the intestinal injury in animal experiments. Overall, our results demonstrated that IL-9 is involved in the damage of the intestinal mucosal barrier in sepsis.

## Methods

### Human patients and controls

Thirty-six adult septic patients (age 18–70 years) admitted into the intensive care unit (ICU) of Nanjing First Hospital were included in this study. The diagnostic criteria for sepsis were in accordance with the surviving sepsis guidelines [1]. Patients with ileus or digestive tract hemorrhage, inflammatory bowel disease, severe abdominal hypertension, chronic organ dysfunction (e.g., hepatic or renal dysfunction), malnutrition, or immunodeficiency, and patients with a history of long-term use of hormones were excluded. All patients received standard treatments for sepsis as needed, including antimicrobial therapy, fluid resuscitation, vasopressor administration, oxygen administration, mechanical ventilation, glucose control, and renal replacement therapy.

Fifteen baseline data (including age, sex, body-mass index, disease severity scores) matched adult non-septic critical patients of our ICU were selected as controls. This study protocol was approved by the Institutional Ethics Committee of Nanjing First Hospital (Approval Number: KY20180713-01), and informed consent was obtained from each patient’s first-degree relatives according to the Declaration of Helsinki. This study was also registered at ClinicalTrials.gov (ID: NCT03791866). The baseline data, including age, sex, body-mass index, etiology of sepsis, disease categories of controls, Acute Physiology and Chronic Health Evaluation II (APACHE II) scores, and Sequential Organ Failure Assessment (SOFA) scores for both septic and control patients, were collected. The IL-9-producing CD4(+) T cell percentages, IL-9, and D-lactate (biomarker of mucosal barrier function) [5] levels in peripheral blood were tested on days 1, 3, and 7 after admission. In addition, clinical outcome variables including 28-day mortality and ICU duration (days) were recorded.

### Sepsis model and groups

To verify the precise roles of IL-9 in the intestinal injury of sepsis, we performed the pre-clinical experiments using SD rats. Adult SD rats (weight 180–220 g, about 6–8 weeks old) were purchased from the experimental animal center of Zhejiang Academy of Medical Sciences (Zhejiang, China). Sepsis was induced by cecum ligation and puncture (CLP) as reported in previous studies [16, 17]. The rats were anesthetized by intraperitoneal injection of 10% chloral hydrate at a dose of 0.3 ml/100 g. An incision of 2.5 cm from middle abdomen was performed under sterile conditions. The cecum was ligated in the middle segment with 3-0 silk and then was punctured with a 22-gauge needle. After that, the cecum was placed back into the abdominal cavity, and the abdomen was closed. Sham-operated (control) rats received identical laparatomy, where the cecum was exposed without ligature or puncture. Normal saline and antibiotic were administered to prevent hypotension.

Rats were randomly assigned to the four groups (*n* = 12 in each group). In addition to the sepsis group and control group (sham operation group), we also added the sepsis+sh-IL-9 (IL-9 inhibition) group and sepsis+IL-9 (IL-9 overexpression) group in the animal experiments. These groups were necessary to observe the effects of IL-9 on the intestinal injury of sepsis from various perspectives. Rats in the sepsis+sh-IL-9 group received adeno-associated virus (AAV) of IL-9 interference, whereas the sepsis+IL-9 group received AAV of IL-9 overexpression by intraperitoneal injection. The AAV was produced according to a previous procedure [18]. Briefly, IL-9 shRNA cassette or cDNA clone fragment encoding IL-9 were constructed into rAAV plasmid, respectively (Genepharma, Shanghai, China). Then the plasmid was co-transfected with AAV package and shuttle plasmids into AAV-293 cell line. The AAV virus particle was harvested after using ammonium sulfate precipitation and iodixanol continuous gradient centrifugation. Genome titers of the AAV particles were determined by real-time polymerase chain reaction. The AAV particle was stored in − 80 °C refrigerator. The dosage of AAV particle was 100 μl, and the titers were 2 × 10^11^ vg/ml [18]. Sepsis was induced 7 days after AAV injection.

Rats were sacrificed on the 7th day after sepsis induction. The rats were anesthetized by intraperitoneal injection of 10% chloral hydrate at a dose of 0.3 ml/100 g before taking serum and intestinal tissue. Blood was collected in tubes with heparin after cardiac puncture, centrifuged (10 min, 1500×*g*). Colons were collected from the cecum to the anus, and some of the intestinal tissue was cut into approximately 4 mm lengths. The tissue was embedded in paraffin and fixed with 4% buffered paraformaldehyde for 12 to 24 h.

### Survival rate analysis

The rats were also divided into four groups: control group, sepsis group, sepsis+sh-IL-9 group, and sepsis+IL-9 group (*n* =10 in each group). After sepsis induction by CLP, rats in these groups were observed and recorded daily for 14 days to assess the survival rate.

### Cytokine and flow cytometry measurement

The human serum concentrations of IL-9 and D-lactate were measured by human enzyme-linked immunosorbent assay (ELISA) kits. The IL-9-producing CD4(+) T cells proportions of serum or intestinal tissue were measured by flow cytometry [FITC-CD4 antibody (eBioscience, 11-0400-82), IL-9 antibody (eBioscience, PA5-47584), Donkey anti-sheep antibody alexa fluor647 (abcam, ab150179)]. The serum was collected from the rats and the concentrations of IL-9 (Cusabio, Wuhan, China) and D-lactate (MyBioSource, San Diego, CA, USA) were also measured by ELISA kits according to the manufacturer’s instructions.

### Western blot

For immunoblotting, the intestinal tissue was lysed in radio immunoprecipitation assay lysis buffer. The lysate was separated in sodium dodecyl sulfate-polyacrylamide gel electrophoresis and transferred to a nitrocellulose membrane. The protein concentration of lysate was detected using a bicinchoninic acid kit (Bio-Rad Laboratories, Hercules, CA, USA). The blot of lysates was incubated with the following antibodies: IL-9 antibody (Abcam, diluted in 1:1000), D-lactate antibody (abcam, diluted in 1:1000), zonula occludens 1(ZO-1) antibody (proteintech, diluted in 1:1000), horseradish peroxidase (HRP)-labeled anti-rabbit antibody (Beyotime, Shanghai, China), GAPDH antibody (ZEN-bio, diluted in 1:5000), and HRP-labeled anti-mouse antibody (Beyotime, Shanghai, China). Images were acquired with Tanon enhanced chemiluminescence substrate (Tanon, Shanghai, China), using Tanon 5200 imaging system (Tanon, Shanghai, China).

### Histopathology

Following rat sacrifice, the intestinal tissue was collected and fixed in 4% formalin and embedded in paraffin. Sections of tissue (4 μm) were stained with hematoxylin and eosin (HE) and observed by Nikon H550S microscope and Nikon DS-Ri2 imaging system (Nikon, Japan). The Chiu score was used to assess the intestinal injury.

### Apoptosis

Apoptosis of intestinal epithelial cells was determined by terminal deoxynucleotidyltransferase-mediated deoxyuridine triphosphate nick-end labeling (TUNEL) assay. Intestine tissue samples were fixed in 4% buffered paraformaldehyde, embedded and cut into 4 μm sections. Then the sections were stained with TUNEL kit (KeyGenBiotech, Nanjing, China) according to the manufacturer’s instructions. Each section was observed under a light microscope and the cells in four randomly selected regions were counted. The positive apoptotic nuclei were brown in color.

### Intestinal permeability

Intestinal mucosal permeability was measured by fluorescence tracing method. A section of intestine about 15 cm long was chosen and ligated at one end, and then the tracer (EZ-Link NHS-Biotin) (Thermo Fisher Scientific, MA, USA) was diluted to 2 mg/ml and injected into the distal intestinal lumen slowly. A segment of intestine about 5 cm long was taken and fixed in 4% paraformaldehyde solution for 3 h, then washed with phosphate buffer solution 3 times and sliced. The tissue was incubated with streptavidin at room temperature for 30 min. The distribution of the fluorescent tracer in intestinal tissue was observed by a laser confocal microscope.

### Statistical analysis

The Kolmogorov-Smirnov test was first performed to test the normal distribution of the data. Normally distributed data were expressed as the means ± standard deviation and were compared by *t* tests. Non-normally distributed data were expressed as the medians (interquartile ranges) and were compared by the Mann-Whitney *U* test or the Kruskal-Wallis test. Categorical variables were presented as absolute numbers or percentages and were analyzed using the *χ*^2^ test or Fisher’s exact test. To take into account the repeated nature of the variables, analysis of variance (ANOVA) and LSD post hoc tests for repeated measurements of the general linear model were implemented. Scatterplots and a correlation analysis were performed to evaluate the relevance between IL-9-producing CD4(+) T cells, IL-9, and D-lactate. Receiver operating characteristic (ROC) curves were used to evaluate the associations between IL-9-producing CD4(+) T cells, IL-9, and 28-day mortality in patients. Survival curves for up to 14 days after sepsis induction of rats were generated using the Kaplan-Meier method and were compared by the log-rank test. IBM Statistical Package for the Social Sciences (SPSS, version 22.0, NY, USA) software was used for statistical analysis, and two-sided *P* < 0.05 was considered statistically significant. The statistical methods of this study were reviewed by Qiao Liu, a biostatistician from the Center for Disease Control and Prevention of Jiangsu Province in China.

## Results

A total of 36 septic patients and 15 non-septic controls were enrolled in this study. The demographic data and clinical parameters of the patients on admission are presented in Table 1. The 28-day mortality was 27.8% (10/36) in these septic patients. Thirteen patients (13/36, 36.1%) received continuous renal replacement therapy, and 33 patients (33/36, 91.7%) received mechanical ventilation during hospital stay.

**Table 1:** Demographic data and clinical parameters on admission

	Sepsis (*n*= 36)		Control (*n*= 15)		*P* value
Age (years)	67.00 (63.25–72.75)		65.20 (59.00–69.25)		0.267
Sex (male:female)	20:16		9:6		0.770
Disease categories (*n*, %)	Etiology of sepsis		Etiology of ICU admission		
	Abdominal infection	19 (52.8%)	Multiple trauma	4 (26.7%)	/
	Thoracic/pulmonary infection	6 (16.7%)	Acute cardiac dysfunction	3 (20.0%)	/
	Blood stream infection	4 (11.1%)	Cerebral hemorrhage	3 (20.0%)	/
	Urinary infection	3 (8.3%)	Acute kidney injury	2 (13.3%)	
	Mucocutaneous infection	2 (5.6%)	ARDS	2 (13.3%)	/
	Other	2 (5.6%)	Hemorrhagic shock	1 (6.7%)	/
BMI (kg/m^2^)	24.22 (21.05–26.02)		24.15 (20.83–25.80)		0.852
APACHE II	23.00 (19.00–27.50)		24.50 (18.50–29.00)		0.632
SOFA	9.50 (8.00–11.75)		11.00 (9.50–13.00)		0.113
ICU days	9.00 (5.25–13.00)		8.50 (5.50–12.75)		0.323
Death (*n*, %)	10 (27.8%)		3 (20.0%)		0.730

*ICU* intensive care unit, *ARDS* acute respiratory distress syndrome, *BMI* body mass index, *APACHE II* Acute Physiology and Chronic Health Evaluation II, *SOFA* Sequential Organ Failure Assessment

**Fig. 1:**
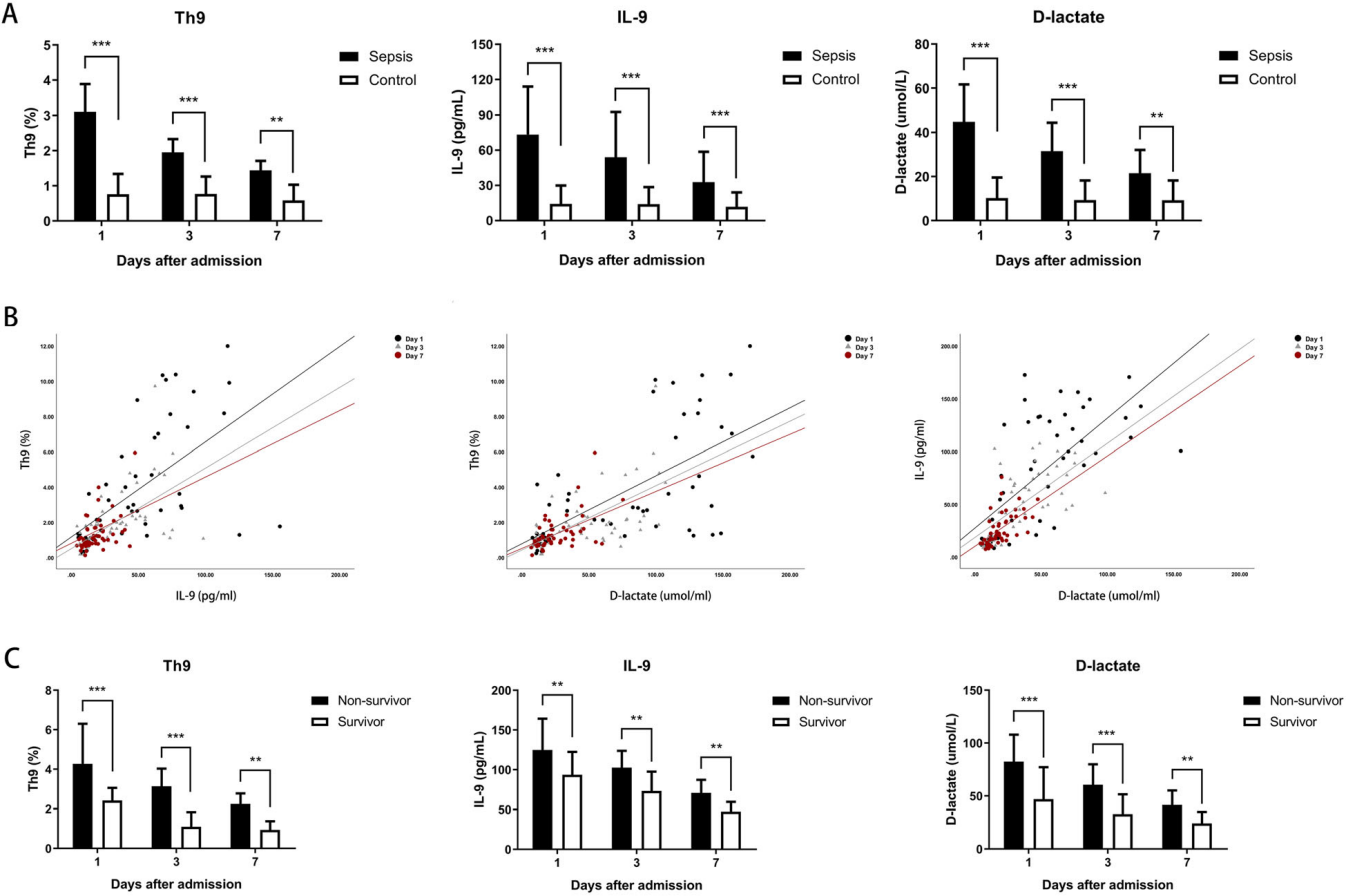
**a** The serum IL-9-producing CD4(+) T cells (Th9) percentages, IL-9, and D-lactate levels of patients in sepsis and control group. **b** The correlations among Th9 percentages, IL-9 levels, and D-lactate levels. IL-9 levels were positively correlated with Th9 percentages on day 1 (*R*^2^ =0.416, *P* < 0.001), day 3 (*R*^2^ =0.351, *P* < 0.001), and day 7 (*R*^2^ =0.305, *P* < 0.001) of admission. IL-9 levels were also positively correlated with D-lactate levels on day 1 (*R*^2^ =0.486, *P* < 0.001), day 3 (*R*^2^ =0.443, *P* < 0.001), and day 7 (*R*^2^ =0.337, *P* < 0.001). Th9 percentages were only positively correlated with D-lactate levels on day 1 (*R*^2^ =0.366, *P* < 0.001). **c** The serum Th9 percentages, IL-9, and D-lactate levels in survivors and non-survivors. * *P* < 0.05, ** *P* < 0.01, *** *P* < 0.001

**Fig. 2:**
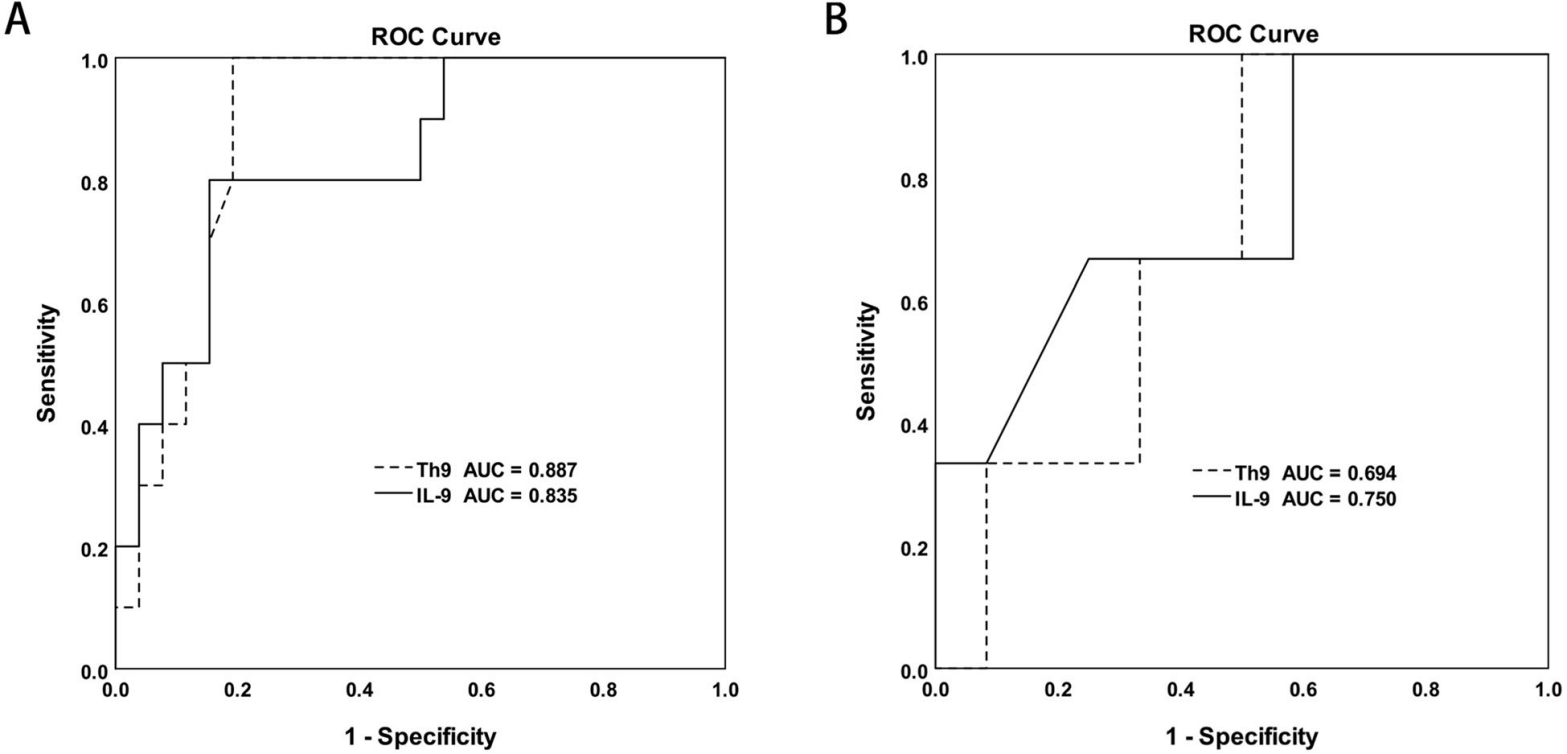
Receiver operating characteristic (ROC) curves were used to evaluate the value of IL-9-producing CD4(+) T cells (Th9) percentages and IL-9 levels on day 1 of admission in predicting 28-day mortality in septic (**a**) and non-septic (**b**) patients. A. The area under the curves (AUCs) of Th9 percentages and IL-9 levels were 0.887 (*P* < 0.001) and 0.835 (*P* =0.002), respectively. **b** The AUCs of Th9 percentages and IL-9 levels were 0.694 (*P* =0.312) and 0.750 (*P* =0.194), respectively

The serum of the septic patients had IL-9-producing CD4(+) T cell percentages, IL-9, and D-lactate levels that were significantly higher than those of the non-septic controls during 7 days of ICU admission (Fig. 1a, *P* < 0.01). The results of scatterplots and correlation analyses were shown in Fig. 1b. IL-9 levels were positively correlated with IL-9-producing CD4(+) T cell percentages on day 1 (*R*^2^ =0.416, *P* < 0.001), day 3 (*R*^2^ =0.351, *P* < 0.001), and day 7 (*R*^2^ =0.305, *P* < 0.001) of admission. IL-9 levels were also positively correlated with D-lactate levels on day 1 (*R*^2^ =0.486, *P* < 0.001), day 3 (*R*^2^ =0.443, *P* < 0.001), and day 7 (*R*^2^ =0.337, *P* < 0.001). Moreover, IL-9-producing CD4(+) T cell percentages were only positively correlated with D-lactate levels on day 1 (R^2^ =0.366, *P* < 0.001). In addition, non-surviving septic patients had significantly higher serum IL-9-producing CD4(+) T cell percentages, IL-9, and D-lactate levels compared with survivors (Fig. 1c, *P* < 0.01). ROC curve results demonstrated that both IL-9-producing CD4(+) T cell percentages and IL-9 levels on day 1 of admission had a high predictive value of 28-day mortality in septic patients (Fig. 2a). The area under the curves (AUCs) of IL-9-producing CD4(+) T cell percentages and IL-9 levels were 0.887 (*P* < 0.001) and 0.835 (*P* =0.002), respectively. The IL-9-producing CD4(+) T cell percentages (AUC =0.694, *P* =0.312) and IL-9 levels (AUC =0.750, *P* =0.194) of non-septic patients had no significant predictive value of 28-day mortality (Fig. 2b). The results suggested that IL-9-producing CD4(+) T cells and IL-9 are associated with prognosis and damage to the intestinal mucosal barrier in septic patients.

**Fig. 3:**
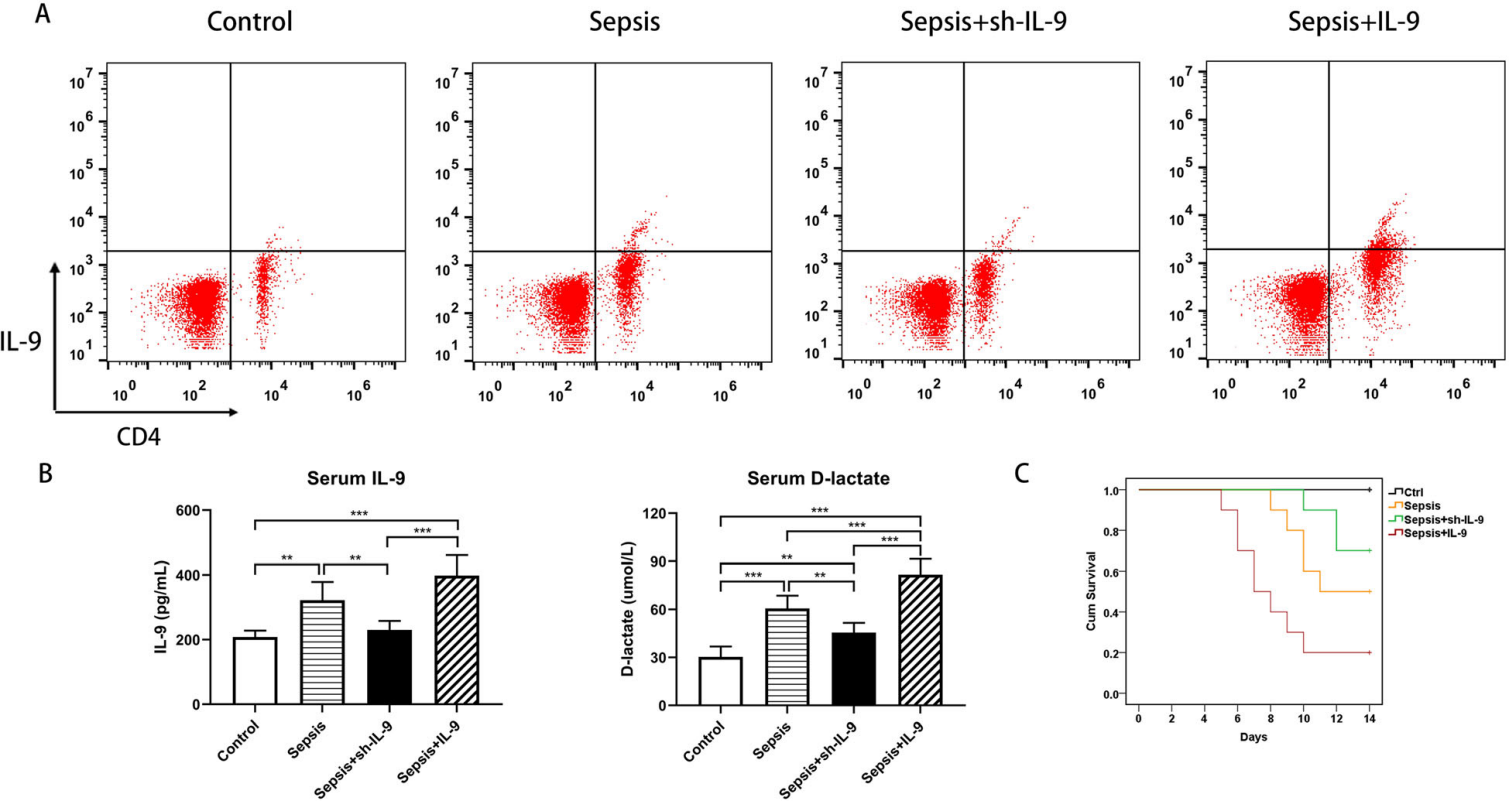
**a** The serum percentages of IL-9-producing CD4(+) T cells of rats in control group (0.43%±0.03%), sepsis group (1.58%±0.41%), sepsis+sh-IL-9 group (0.80%±0.02%), and sepsis+IL-9 group (3.53%±0.90%), respectively. The right upper quadrant of each figure represents a subset of IL-9-producing CD4(+) T cells. **b** The serum IL-9 and D-lactate levels among the four groups. **c** Survival curves for up to 14 days of rats among the four groups (*P* < 0.01). * *P* < 0.05, ** *P* < 0.01, *** *P* < 0.001

To functionally confirm the role of IL-9-producing CD4(+) T cells and IL-9 in the intestinal pathology, we used a rat sepsis model. A total of 48 SD rats were randomly assigned to four groups as follows: control group, sepsis group, sepsis+sh-IL-9 group, and sepsis+IL-9 group. As shown in Fig. 3a, the serum percentages of IL-9-producing CD4(+) T cells of the control group were significantly lower than that of the sepsis group (0.43%±0.03% vs. 1.58%±0.41%, *P* =0.021) or the sepsis+IL-9 group (0.43%±0.03% vs. 3.53%±0.90%, *P* < 0.001). The serum percentages of IL-9-producing CD4(+) T cells of the sepsis+IL-9 group were significantly higher than that of the sepsis group (3.53%±0.90% vs. 1.58%±0.41%, *P* =0.001) or the sepsis+sh-IL-9 group (3.53%±0.90% vs. 0.80%±0.02%, *P* < 0.001). The serum IL-9 and D-lactate levels in the control group were significantly lower than that of the sepsis group and sepsis+IL-9 group (Fig. 3b, *P <* 0.01*)*. Moreover, the serum levels of the two markers were gradually increased in the sepsis+sh-IL-9 group, sepsis group, and sepsis+IL-9 group (Fig. 3b, *P <* 0.01*)*. Survival rate analysis also showed that the survival rate of rats was gradually decreased in the control group (100%), the sepsis+sh-IL-9 group (70%), the sepsis group (50%), and the sepsis+IL-9 group (20%) (Fig. 3c, *P* < 0.001).

**Fig. 4:**
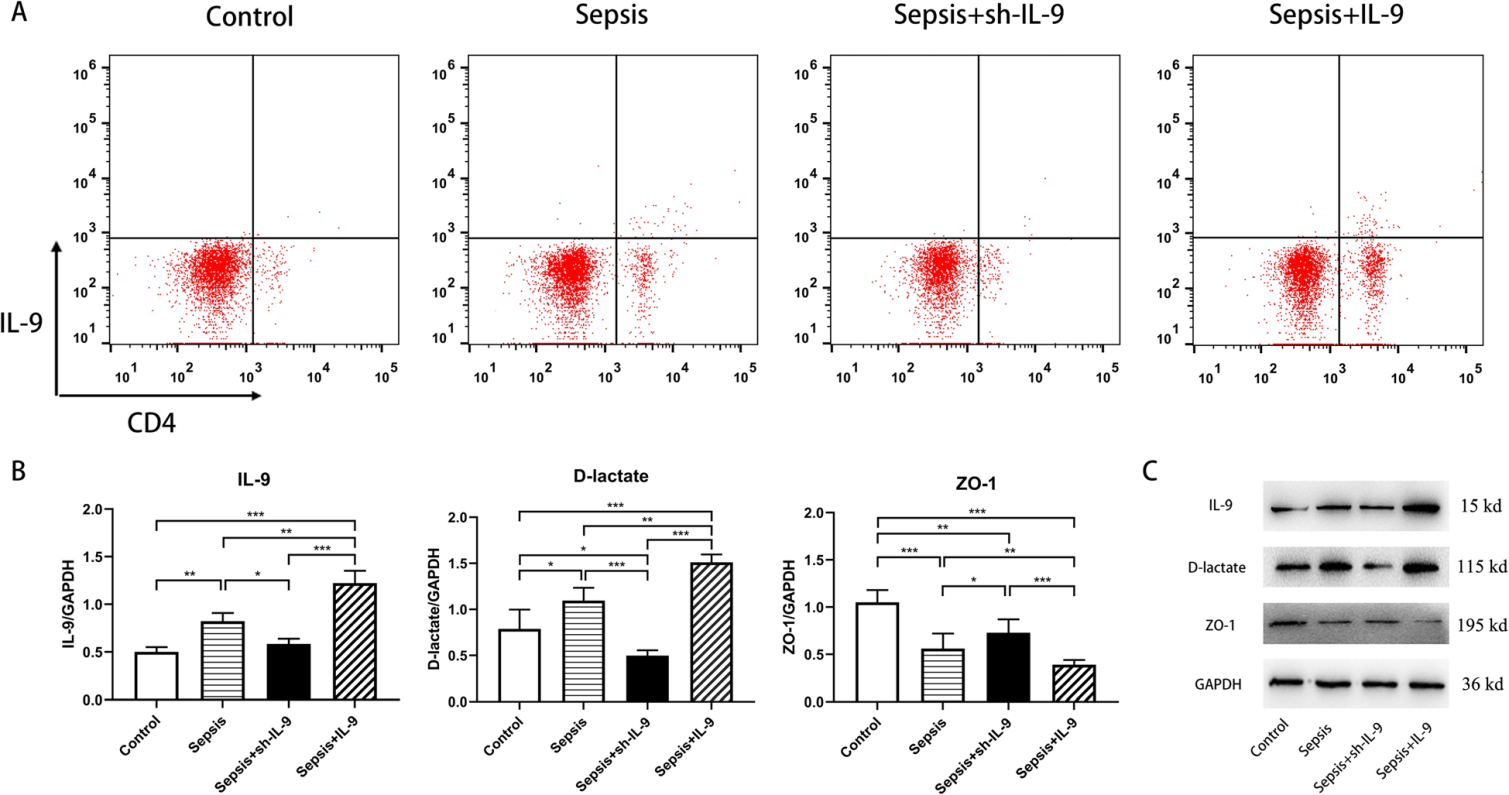
**a** The intestinal tissue percentages of IL-9-producing CD4(+) T cells of rats in control group (0.22%±0.04%), sepsis group (1.02%±0.07%), sepsis+sh-IL-9 group (0.16%±0.01%), and sepsis+IL-9 group (1.57%±0.10%), respectively. The right upper quadrant of each figure represents a subset of IL-9-producing CD4(+) T cells. **b**, **c** The expression levels of IL-9, D-lactate, and zonula occludens 1 (ZO-1) in intestinal tissue of rats among the four groups (*n* =6 in each group). * *P* < 0.05, ** *P* < 0.01, *** *P* < 0.001

The differences of IL-9-producing CD4(+) T cell percentages, IL-9, and D-lactate levels in intestinal tissue of rats among the four groups were shown in Fig. 4. As shown in Fig. 4a, the percentages of tissue IL-9-producing CD4(+) T cells in the control group were significantly lower than that in the sepsis group (0.22%±0.04% vs. 1.02%±0.07%, *P* < 0.001) or the sepsis+IL-9 group (0.22%±0.04% vs. 1.57%±0.10%, *P* < 0.001). The percentages of tissue IL-9-producing CD4(+) T cells in the sepsis group were significantly higher than that in the sepsis+sh-IL-9 group (1.02%±0.07% vs. 0.16%±0.01%, *P* < 0.001), whereas IL-9-producing CD4(+) T cell percentages significantly lower than that in the sepsis+IL-9 group (1.02%±0.07% vs. 1.57%±0.10%, *P* < 0.01). Western blot analysis (Fig. 4b, c) showed that the expression levels of IL-9 and D-lactate in intestinal tissue of controls were significantly lower than that of the sepsis group (*P* < 0.05) and the sepsis+IL-9 group (*P* < 0.001). Compared with the sepsis group, the expression levels of IL-9 and D-lactate were significantly increased in the sepsis+IL-9 group (*P* < 0.01), whereas those were significantly decreased in the sepsis+sh-IL-9 group (*P* < 0.001). The ZO-1 levels of intestinal tissue in controls were significantly higher than that of the other three groups (Fig. 4b, c, *P* < 0.001). Compared with the sepsis group, the ZO-1 levels were significantly increased in the sepsis+sh-IL-9 group (*P* < 0.01), whereas those were significantly decreased in the sepsis+IL-9 group (*P* < 0.001).

**Fig. 5:**
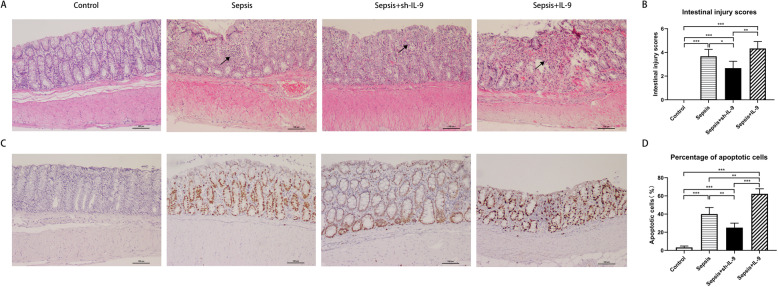
**a** The hematoxylin and eosin stain of intestinal mucosa in rats of the control group, sepsis group, sepsis+sh-IL-9 group, and sepsis+IL-9 group, respectively. **b** The intestinal injury scores of rats among the four groups. **c** The epithelial cells apoptosis detection of intestinal mucosa in rats among the four groups. **d** The percentages of apoptotic cells of intestinal mucosa in rats among the four groups. * *P* < 0.05, ** *P* < 0.01, *** *P* < 0.001

HE staining (Fig. 5a) showed that intestinal mucosa was normal, and the epithelium was intact in non-septic controls. In the sepsis group, lamina propria hemorrhage and inflammatory cell infiltration were also observed. The sepsis-induced intestinal mucosal injuries were significantly improved or aggravated by IL-9 interference or IL-9 overexpression, respectively (Fig. 5a). Morphological injuries were assessed by Chiu’s score (Fig. 5b, *P* < 0.05). TUNEL apoptosis detection also demonstrated similar findings (Fig. 5c). The positive apoptotic nuclei were brown in color. The percentage of apoptotic cells in intestinal mucosa was gradually increased in the control group, sepsis+sh-IL-9 group, sepsis group, and sepsis+IL-9 group (Fig. 5d, *P* < 0.01).

**Fig. 6:**
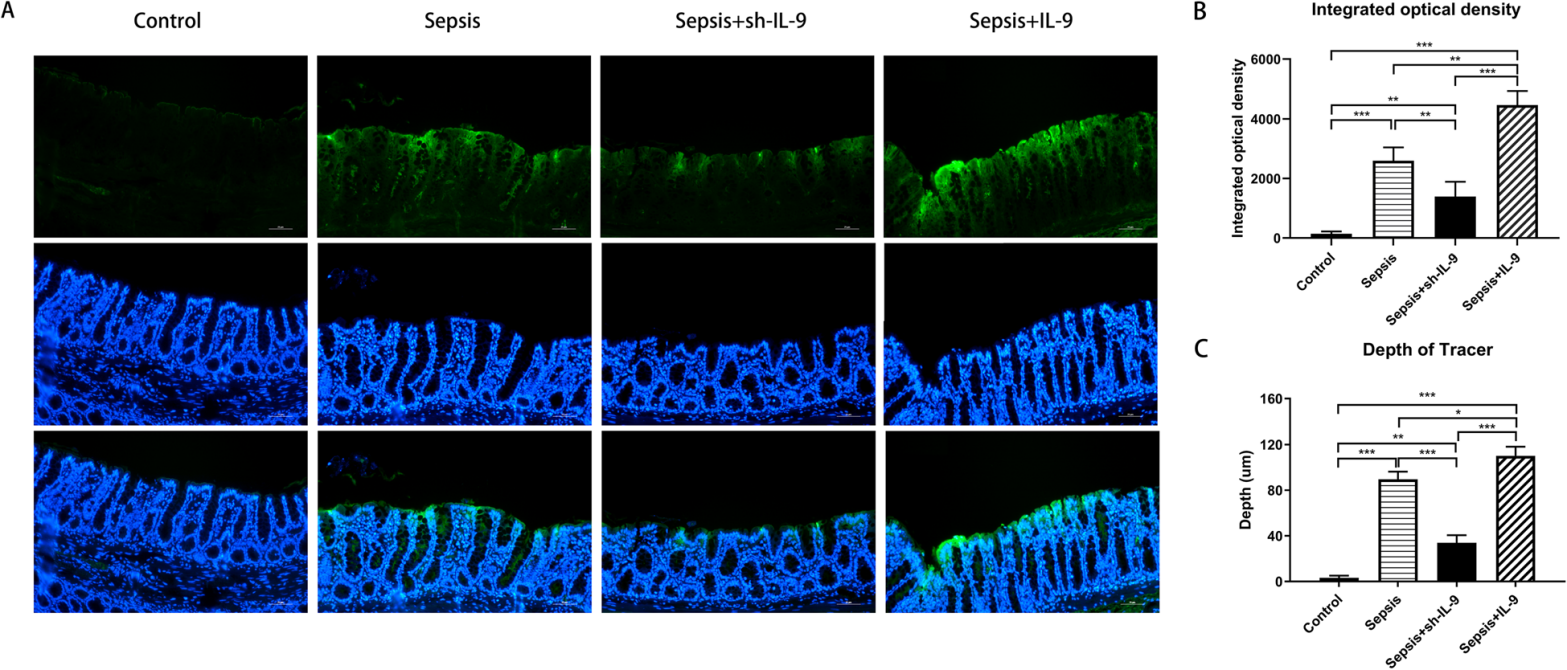
**a** Detection of intestinal mucosal permeability in rats of the control group, sepsis group, sepsis+sh-IL-9 group, and sepsis+IL-9 group, respectively. **b** The integrated optical density (IOD) values of fluorescent staining in the intestinal mucosa of rats among the four groups. **c** The penetration depth of tracer in the intestinal mucosa of rats among the four groups. * *P* < 0.05, ** *P* < 0.01, *** *P* < 0.001

Intestinal mucosal permeability was detected by a laser confocal microscope (Fig. 6). The distribution of fluorescent tracer (green color) was observed only on the surface of intestinal mucosa in non-septic controls (Fig. 6a). In the sepsis group, the tracer penetrated into the muscular layer of the intestinal mucosa (Fig. 6a). After IL-9 interference, the tracer only penetrated into the epithelial layer of intestinal mucosa, while after IL-9 overexpression, the tracer penetrated deeply into the intestinal submucosa (Fig. 6a). Comparisons of integrated optical density (IOD) values were shown in Fig. 6b. The IOD values of fluorescent tracer were gradually increased in the control group, sepsis+sh-IL-9 group, sepsis group, and sepsis+IL-9 group (*P* < 0.01). Moreover, we also compared the differences of tracer penetration depth among these groups. The penetration depth of tracer was gradually increased in the control group, sepsis+sh-IL-9 group, sepsis group, and sepsis+IL-9 group (Fig. 6c, *P* < 0.05).

## Discussion

It is known that IL-9-producing CD4(+) T cells and IL-9 are associated with inflammatory bowel disease, tumor, and autoimmune diseases [7, 9, 10]. However, the roles of IL-9-producing CD4(+) T cells and IL-9 in sepsis are not clear. Intestinal mucosal barrier injury is one of the important potentiators of sepsis [5, 6]. This study firstly proved that IL-9-producing CD4(+) T cells and IL-9 were also closely related to the barrier injury and mortality in sepsis. We found that the serum IL-9-producing CD4(+) T cell percentages, IL-9, and D-lactate levels were significantly higher in septic patients or rats than that in controls. IL-9-producing CD4(+) T cell percentages and IL-9 levels were positively correlated with D-lactate levels in septic patients. IL-9-producing CD4(+) T cell percentages and IL-9 levels also had a high predictive value of 28-day mortality. The non-survivors had significantly higher serum IL-9-producing CD4(+) T cell percentages, IL-9, and D-lactate levels compared with survivors. In septic rats, IL-9 increased the expression levels of D-lactate, whereas that decreased the expression levels of ZO-1. Moreover, the barrier injury was aggravated or alleviated by increasing or interfering with IL-9 expression, respectively. Survival rate analysis also showed that IL-9 decreased the 14-day survival rate of septic rats.

The intestinal tract is considered a vital immune organ. Acute intestinal barrier damage and systemic infection are a vicious cycle in critical diseases, especially in sepsis [1, 19, 20]. Our previous studies showed that T helper lymphocytes were associated with gastrointestinal injury and immune dysregulation in sepsis [20, 21]. In addition to the conventional Th1/Th2 and Th17/Treg lymphocytes, Th9 cells also contribute to chronic intestinal and airway inflammation [7–10, 12, 14]. Renga et al. [8] reported that IL-9 and mucosal mast cells contributed to barrier function loss, dissemination, and inflammatory dysbiosis in experimental leaky gut models and human celiac disease. Feng et al. [9] found that serum IL-9 level was upregulated significantly and correlated with disease severity in patients with Crohn’s Disease. Wang [14] and Zhao’s [22] research showed that tumor necrosis factor-like ligand 1A could destroy the mucosal barrier by promoting Th9 and IL-9 overexpression in mice with chronic colitis. Consequently, it is reasonable to speculate that Th9 and IL-9 may be related to the barrier injury in sepsis. Our findings suggest that IL-9 overexpression can increase inflammatory cell infiltration, epithelial cell apoptosis, and mucosal permeability of the intestine in septic rats. D-lactate and ZO-1 are common biomarkers of mucosal barrier function [17, 22]. The D-lactate level was positively correlated with the degree of intestinal barrier injury, whereas the ZO-1 was opposite [5, 6]. In both animal models and humans of our study, IL-9-producing CD4(+) T cell percentages and IL-9 levels were positively correlated with the concentrations of D-lactate. In animal models, IL-9 increased the expression level of D-lactate, whereas that decreased the expression level of ZO-1. These results suggest that IL-9-producing CD4(+) T cells and IL-9 could be potential therapeutic targets of intestinal mucosal injury in sepsis. Our findings also support this conclusion, because the mucosal injury was improved after IL-9 interference in septic rats.

Intestinal mucosal barrier damage and enterogenous infection contribute to the high mortality in sepsis [1, 3]. In other words, non-surviving septic patients tend to have more severe barrier damage compared with survivors. In our study, the non-survivors had significantly higher serum IL-9-producing CD4(+) T cell percentages, IL-9, and D-lactate levels compared with survivors. ROC curve results demonstrated that both IL-9-producing CD4(+) T cell percentages and IL-9 levels had a high predictive value of 28-day mortality. Survival rate analysis of animal models also showed that IL-9 decreased the 14-day survival rate of sepsis rats. Therefore, inhibition of Th9 cells differentiation or IL-9 expression may be a novel strategy to improve survival in sepsis. We are now conducting a clinical study of this project (not yet published). Th9 cells are derived from naive T cells in the presence of transforming growth factor β and interleukin-4 [7, 10]. In addition to IL-9, Th9 cells also express IL-10, IL-21, and other cytokines [10]. Interestingly, we found that Th9 percentages of serum and intestinal tissue were decreased after IL-9 interference, whereas IL-9-producing CD4(+) T cell percentages increased after IL-9 overexpression in septic rats. The phenomenon revealed that IL-9-producing CD4(+) T cells and IL-9 might have a feedback relationship through some potential pathways, but the mechanism was not clear and remained to be tested by further research.

Although numerous studies have elucidated the role of IL-9 in pro-inflammatory activity, some studies found that IL-9 also had anti-inflammatory effects. IL-9 could protect mice from Gram-negative bacterial shock by suppressing TNF-alpha, IL-12, and IFN-gamma, or inhibit oxidative burst and TNF-alph release in lipopolysaccharide-stimulated human monocytes [13, 23, 24]. In the present study, we found that IL-9 mainly played a pro-inflammatory role in the intestinal mucosal injury of sepsis. IL-9 was associated with epithelial cell apoptosis and increased mucosal permeability in septic rats. However, whether IL-9-producing CD4(+) T cells and IL-9 have protective effects in sepsis need confirmation by more studies.

*Some limitations* of this study should be discussed. Due to our single-center design and small sample size, the results may not be generalizable, and the accuracy should be confirmed by large-scale clinical prospective studies. Moreover, Th9 cells can secrete a variety of cytokines, and IL-9 is also secreted by other cells. The roles of these incidental cytokines or cells were not investigated in our study. In addition, only the IL-9 pathway instead of the IL-9-producing CD4(+) T cells was intervened in this study. The specific mechanism by which IL-9-producing CD4(+) T cells are involved in the mucosal barrier injury of sepsis needs further research.

## Conclusions

This study suggested that IL-9 is closely related to the intestinal mucosal barrier injury in sepsis. Patients with sepsis had increased IL-9 levels compared to patients without sepsis. IL-9-producing CD4(+) T cells and IL-9 levels were positively correlated with D-lactate levels and had a high predictive value of 28-day mortality in septic patients. The intestinal barrier injury was aggravated or alleviated by increasing or interfering with the expression of IL-9, respectively. IL-9 also increased the mortality of sepsis in animal models. Further studies are warranted to investigate whether IL-9 blockade has the potential to improve the mucosal barrier injury in sepsis.

## Data Availability

Not applicable.

## Abbreviations

Th: T helper
Treg: T regulatory
Th9: IL-9-producing CD4(+) T helper
IL-9: Interleukin-9
ICU: Intensive care unit
APACHE II: Acute Physiology and Chronic Health Evaluation II
SOFA: Sequential Organ Failure Assessment
CLP: Cecum ligation and puncture
AAV: Adeno-associated virus
ELISA: Enzyme-linked immunosorbent assay
ZO-1: Zonula occludens 1
HRP: Horseradish peroxidase
HE: Hematoxylin and eosin
TUNEL: Terminal deoxynucleotidyltransferase-mediated deoxyuridine triphosphate nick-end labeling
ANOVA: analysis of variance
ROC: Receiver operating characteristic
SPSS: Statistical Package for the Social Sciences
